# Regulation Mechanisms of the Glutamate Transporter in the Response of Pacific Oyster upon High-Temperature Stress

**DOI:** 10.3390/ijms252111342

**Published:** 2024-10-22

**Authors:** Xueshu Zhang, Xue Wen, Yiran Si, Deliang Li, Chuanyan Yang, Lingling Wang, Linsheng Song

**Affiliations:** 1 Liaoning Key Laboratory of Marine Animal Immunology & Disease Control, Dalian Ocean University, Dalian 116023, China; 2 Southern Marine Science and Engineering Guangdong Laboratory (Zhuhai), Zhuhai 519000, China; 3 Liaoning Key Laboratory of Marine Animal Immunology, Dalian Ocean University, Dalian 116023, China; 4 Dalian Key Laboratory of Aquatic Animal Disease Prevention and Control, Dalian Ocean University, Dalian 116023, China

**Keywords:** Pacific oyster, glutamate transporter, high-temperature stress, ferroptosis

## Abstract

Glutamate transporters (GLTs) are integral to the glutamatergic system, modulating glutamate homeostasis to enhance resilience and resistance against environmental stress. There are six GLTs identified in the Pacific oyster (*Crassostrea gigas*), which were categorized into two subfamilies: excitatory amino acid transporters (*Cg*EAATs) and vesicular glutamate transporters (*Cg*VGLUTs). The *Cg*EAATs harbor a GltP domain, while *Cg*VGLUTs feature an MFS domain, both with conserved sequence and structural characteristics. The expression of *Cg*GLTs is elevated during the planktonic larval stage compared to the fertilized egg stage and is constitutively expressed in various tissues of adult oysters, suggesting its critical role in both larval development and the physiological processes of adult oysters. Transcriptomic analysis revealed diverse expression patterns of GLTs in oyster gills after 7 days of high-temperature stress, with *CgEAAT3* showing a significant upregulation. A KEGG pathway enrichment analysis identified glutathione metabolism and ferroptosis as prominently enriched pathways. At 48 h after high-temperature stress, the expression levels of Glutathione Peroxidase 4 (*CgGPX4*) and *CgEAAT3*, along with elevated Fe content in the gills, significantly increased. Moreover, the RNAi-mediated the inhibition of *CgEAAT3* expression under high-temperature stress, resulting in a significant reduction in *CgGPX4* expression and a further increase in Fe accumulation in oyster gills. These results indicate that *CgEAAT3* contributes to the regulation of ferroptosis and redox homeostasis by modulating *CgGPX4* expression. This study provides new insights into the adaptive mechanisms of bivalves to environmental stress.

## 1. Introduction

Glutamate transporters (GLTs) regulate signal transduction, cellular metabolism, and environmental sensing by facilitating the transport of excitatory glutamate [1]. As members of the solute carrier (SLC) superfamily, GLTs are classified into three subclasses: excitatory amino acid transporters (EAATs), vesicular glutamate transporters (VGLUTs), and the glutamate-cystine exchange transporter (SLC7A11/xCT) [2]. EAATs with a conserved GltP domain facilitate the rapid uptake of glutamate from the extracellular space to prevent neurotoxicity [3,4]. VGLUTs with major facilitator superfamily (MFS) domains manage the storage and release of glutamate from synaptic vesicles, crucial for synaptic transmission [5,6]. SLC7A11/xCT, with its heterodimeric structure of light and heavy subunits, facilitates a non-vesicular glutamate release pathway and contributes to cellular redox balance [2,7,8]. These distinct structural features highlight the specialized roles of these transporters in regulating glutamate levels, supporting neural communication and adaptation to environmental stress.

Glutamate transporters play a critical role in modulating environmental stress responses. In *Caenorhabditis elegans*, the vesicle glutamate transporter promotes glutamate release in neurons under a high temperature, leading to synapse-specific subcellular defects [9]. Existing reports indicate that high-temperature stress enhances glutamate release and exacerbates glutamate-induced neurotoxicity. Glutamate transporters are essential for maintaining low extracellular glutamate concentrations and preventing excitotoxicity [8]. Furthermore, studies in plants have shown that heat-induced cell death resembles ferroptosis [10]. However, it remains unclear whether high temperatures induce ferroptosis in invertebrates and whether this process is regulated by glutamate. This study aims to explore whether glutamate transporters are involved in ferroptosis under heat stress, thereby elucidating their potential roles in adaptive stress responses.

The Pacific oyster (*Crassostrea gigas*) is a key marine resource with significant ecological, economic, and scientific value. As a representative intertidal benthic organism, *C. gigas* is frequently exposed to environmental stresses, particularly seasonal temperature fluctuations that affect its growth, reproduction, and survival [11,12,13]. Understanding the response mechanisms of *C. gigas* to temperature stress is critical for developing strategies to prevent large-scale summer mortality and ensuring population and ecosystem stability [14]. Temperature fluctuations significantly impact the physiology and survival of oysters, with elevated temperatures causing oxidative stress and mass mortality events, and low temperatures suppressing metabolism and immunity, increasing susceptibility to diseases [15,16]. Therefore, understanding thermal tolerance mechanisms in benthic bivalves is crucial [17]. This study identifies and analyzes the GLT family in *C. gigas*, examining their structural classification and spatiotemporal expression under high-temperature stress. Additionally, we analyzed the impact of *Cg*GLTs on iron (Fe) levels and glutathione peroxidase 4 (GPX4) activity during high-temperature stress. This study provides new insights into the role of GLTs in bivalves under high-temperature stress and deepens our understanding of stress responses in mollusks.

## 2. Results

### 2.1. Characterization and Expression Patterns of the Glutamate Transporter Family Genes in C. gigas

By comparing the transcriptome and genome databases of *C. gigas*, six glutamate transporter genes were identified. Based on sequence homology and domain characteristics, these genes were divided into two subfamilies: the vesicular glutamate transporter family (VGLUTs) and the excitatory amino acid transporter family (EAATs), named *CgVGLUT1*, *CgVGLUT2*, *CgVGLUT3*, *CgEAAT1*, *CgEAAT2*, and *CgEAAT3*. The sequence characteristics of the glutamate transporter genes are summarized in Table 1. The open reading frames (ORFs) of the glutamate transporter genes in *C. gigas* range from 1467 bp (*CgVGLUT3*) to 1887 bp (*CgVGLUT1*), encoding 488 to 628 amino acids. *CgVGLUT2* and *CgVGLUT3* have the highest number of exons among all *C. gigas* glutamate transporter genes, consisting of 13 exons and 12 introns (Supplementary Figure S1). The predicted molecular weight of the *C. gigas* glutamate transporter genes ranges from 53.14 to 69.61 kDa, with isoelectric points (pI) ranging from 5.25 to 8.87. The secondary structure analysis of the proteins encoded by *C. gigas* glutamate transporter genes indicates that these proteins are composed of 21 to 30 alpha helices, 31 to 42 beta strands, 29 to 50 coils, and 27 to 51 turns. The calculated hydrophobicity index (GRAVY) averages between 0.129 and 0.564, indicating that they are intrinsically hydrophobic. The amino acid sequence identity with other invertebrates ranges from 33.41% to 60.18%, and with vertebrates from 32.44% to 60.63% (Table 2).

**Figure 1:**
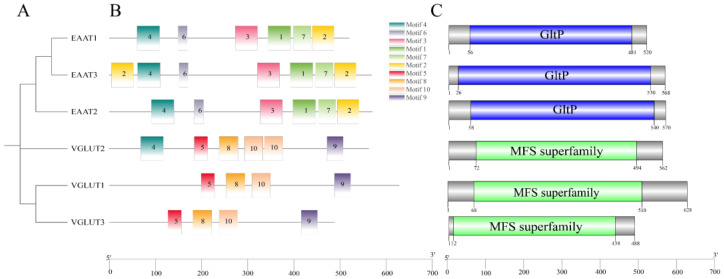
Phylogenetic relationships, motifs, and gene structure of six glutamate transporter genes in Pacific oysters. (**A**) Phylogenetic tree of glutamate transporters. Protein sequences were aligned using AliView, and the phylogenetic tree was constructed using the maximum likelihood method in PhyML3.0. (**B**) Protein motifs of glutamate transporters. Conserved motifs were identified using MEME and visualized with TBtools software (V2.101). Colored boxes represent conserved motifs with different sequences and sizes (1–10). The scale at the bottom estimates the length of each protein. (**C**) Gene structure: blue boxes represent GltP domains, and green boxes represent MFS domains.

A domain analysis showed that all excitatory amino acid transporters contain the GltP domain (Figure 1C). *Cg*VGLUT1 and *Cg*VGLUT3 contain an MFS domain (Figure 1C). Further exploration of motifs in *C. gigas* glutamate transporters revealed ten conserved motifs (Figure 1A,B). *Cg*EAATs have six common motifs: motifs 1, 2, 3, 4, 6, and 7, with *Cg*EAAT3 also containing motif 2. *Cg*VGLUTs share four common motifs: motifs 5, 8, 9, and 10, with *Cg*VGLUT2 containing two copies of motif 10 and an additional motif 4.

### 2.2. Phylogenetic Analysis and Chromosomal Localization of the Glutamate Transporter Family in C. gigas

**Figure 2:**
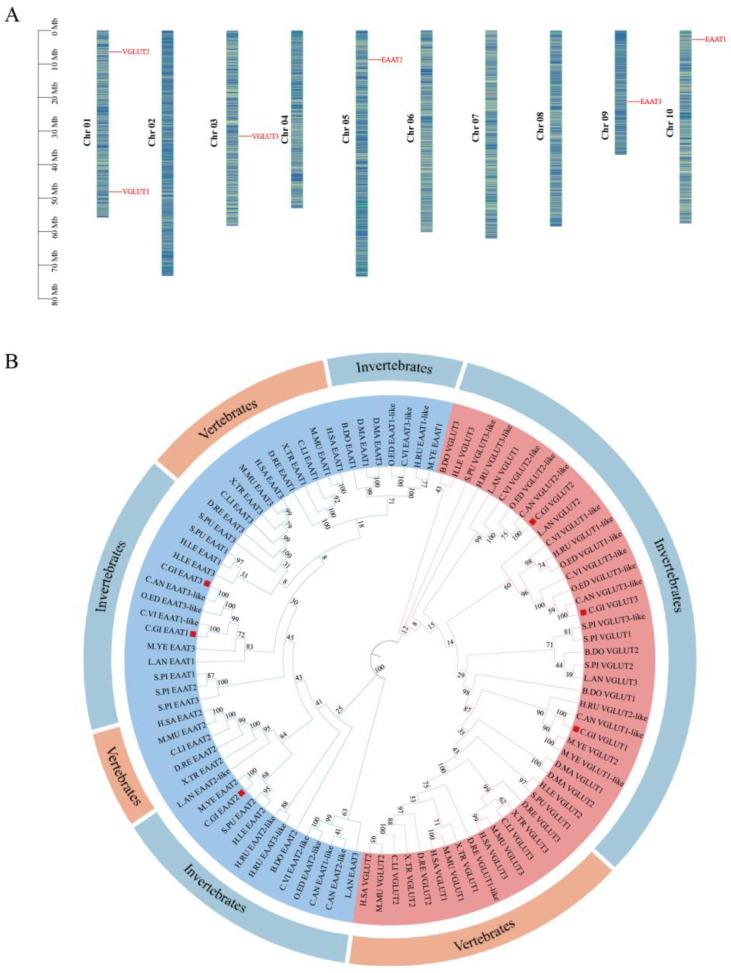
Chromosomal localization and phylogenetic analysis of glutamate transporter genes in Pacific oysters. (**A**) Chromosomal distribution of the six identified glutamate transporter genes across the 10 chromosomes of Pacific oysters. Chromosome numbers (Chr 01–Chr 10) are displayed on the left side of the chromosomes, and gene names are shown on the right side. (**B**) Phylogenetic tree of multiple species: constructed using the neighbor-joining method in MEGA-X, with Pacific oyster glutamate transporters marked by red squares. Different colors represent branches of different subfamilies (*Cg*VGLUT: red; *Cg*EAAT: blue).

The chromosomal map of the glutamate transporter family in *C. gigas* was drawn based on the physical location information from the *C. gigas* genome (Figure 2). The results showed that the six identified glutamate transporter genes are concentrated on chromosomes 1, 3, 5, 9, and 10 of *C. gigas*. Chromosome 1 contains two glutamate transporter genes, while chromosome 5, the longest chromosome, contains only one gene, indicating that the number of glutamate transporter genes on each chromosome is not related to chromosome size.

To explore the phylogenetic relationships of glutamate transporter genes among different species, a phylogenetic tree was constructed using amino acid sequences from various vertebrates and invertebrates (Supplementary Table S1; Figure 2B). The phylogenetic results showed that the glutamate transporters of *C. gigas* are closely related to those of the Portuguese oyster. The glutamate transporter family of *C. gigas* is divided into two branches, representing the EAAT and VGLUT subfamilies. The red branch represents the VGLUT subfamily, where *C. gigas* VGLUT1 clusters with *C. angulata* VGLUT1-like, *C. gigas* VGLUT2 clusters with *C. angulata* VGLUT2-like, further clusters with *O. edulis* VGLUT2-like and *C. livia* VGLUT2-like, and *C. gigas* VGLUT3 clusters with *C. angulata* VGLUT3. The blue branch represents the EAAT subfamily, where *C. gigas* EAAT1 clusters with *C. virginica* EAAT1-like and further clusters with *C. gigas* EAAT3, while *C. gigas* EAAT2 clusters with *M. yessoensis* EAAT2.

### 2.3. Spatiotemporal Expression Characteristics of Glutamate Transporters in C. gigas

**Figure 3:**
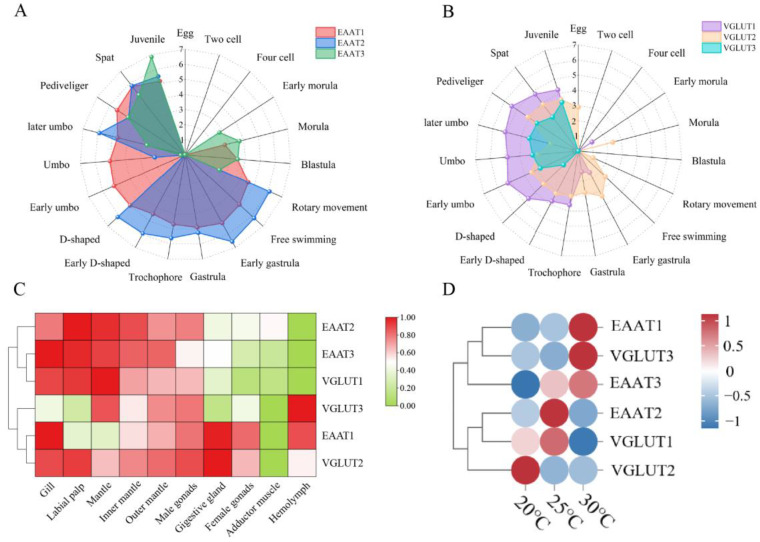
Temporal and spatial expression characteristics of glutamate transporter genes in Pacific oysters and their expression patterns under different temperature stresses. (**A**) Expression profiles of excitatory amino acid transporter genes in Pacific oysters at various developmental stages from egg to larvae. (**B**) Expression profiles of vesicular amino acid transporter genes in Pacific oysters at various developmental stages from egg to larvae. (**C**) Summary of the expression levels of glutamate transporter genes in the gill, labial palps, the mantle, the mantle edge, the outer mantle edge, the male gonad, the digestive gland, the female gonad, the adductor muscle, and the hemolymph cells based on Log_2_RPKM. (**D**) Expression patterns of glutamate transporter genes in the gill tissues of Pacific oysters under different high-temperature stresses.

Using the K-means clustering algorithm, different expression patterns of the six glutamate transporters during oyster development were identified (Figure 3). *CgEAAT1* is continuously expressed from the morula stage to the juvenile stage. *CgEAAT2* is expressed from the rotary movement to the juvenile stage, except in the early umbo stage. *CgEAAT3* shows expression mainly in two stages: it first appears in the late embryonic development stage (early morula to rotary movement) and gradually increases after the start of feeding (D-shaped to juvenile), peaking in the juvenile stage (RPKM = 950, Figure 3A). *CgVGLUTs* are expressed from the D-shaped to the juvenile stages, peaking in the pediveliger stage (RPKM = 102.5, Figure 3B). Among all glutamate transporters, only *CgVGLUT2* is detected in the egg stage, while no glutamate transporters are expressed in the two-cell to four-cell stage. Additionally, *CgEAATs* and *CgVGLUTs* show complementary expression patterns in the blastula and early umbo stages, with *CgEAATs* expressed in the blastula stage and *CgVGLUTs* in the early umbo stage.

The expression patterns of glutamate transporters in adult Pacific oyster tissues can be categorized into two distinct groups (Figure 3C). One group consists of *CgEAAT2*, *CgEAAT3*, and *CgVGLUT1*, which exhibit the lowest expression levels in hemocytes (RPKM = 169) and significantly higher levels in the gills, labial palps, and the mantle (RPKM = 5056.67, 5344, and 4413.92, respectively). The other groups include *CgVGLUT3*, *CgEAAT1*, and *CgVGLUT2*, where *CgVGLUT3* is highly expressed in hemocytes (RPKM = 327), and *CgEAAT1* shows elevated expression in the gill and digestive gland tissues (RPKM = 2571 and 2436, respectively). *CgVGLUT2* demonstrates high expression across all the tissues except the adductor muscle. Additionally, the expression of glutamate transporters is particularly elevated at the inner and outer edges of the mantle (RPKM = 1882.33 and 1688.33, respectively).

### 2.4. Expression of Glutamate Transporters in C. gigas Under High-Temperature Stress

RNA-seq data were used to analyze the expression patterns of GLTs in the gills of *C. gigas* after 7 days of exposure to temperatures from 20 °C to 30 °C, characterizing their response to high-temperature stress (Figure 3D). The data showed that *CgEAAT1* and *CgVGLUT3* have similar expression trends, both peaking at 30 °C. *CgEAAT2* and *CgVGLUT1* exhibit an initial increase followed by a decrease from 20 °C to 30 °C, peaking at 25 °C. Interestingly, *CgEAAT3* and *CgVGLUT2* show opposite expression trends under high temperature stimulation, with *CgEAAT3* increasing and *CgVGLUT2* decreasing as the temperature rises. The GLTs display complex response patterns, with *CgEAAT3* playing a crucial role under high-temperature stress.

### 2.5. Mechanism of CgEAAT3 Response to High-Temperature Stress

**Figure 4:**
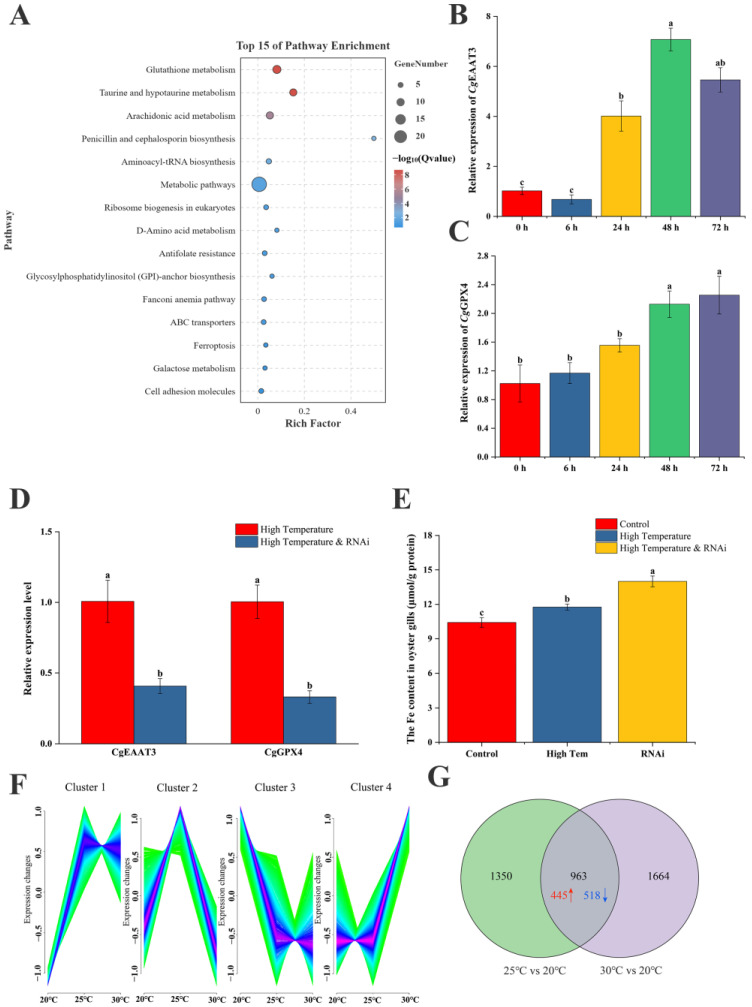
Mechanism of *CgEAAT3* response to high-temperature stress. (**A**) Top 15 KEGG enrichment terms for differentially expressed genes with similar trends to *CgEAAT3*, identified under both 25 °C and 30 °C stress conditions. (**B**) Fold change in expression of the *CgEAAT3* gene under high-temperature stress. (**C**) Fold change in expression of the *CgGPX4* gene under high-temperature stress. (**D**) Fold change in expression of the *CgEAAT3* and *CgGPX4* genes after interference with *CgEAAT3* under high-temperature stress. (**E**) Changes in iron content in gill tissues after interference with *CgEAAT3* under high-temperature stress. (**F**) Temperature trends and clustering of gene expression under different temperature stresses analyzed using Mfuzz. The x-axis represents the three temperatures, and the y-axis represents the normalized intensity ratios (log_2_ transformed) at each stage. (**G**) Differentially expressed genes showing a similar trend to *CgEAAT3* at 25 °C and 30 °C compared to the control group. Red indicates upregulated genes, and blue indicates downregulated genes. Significant differences between groups are indicated by different letters (a, b, c), with statistical significance set at *p* < 0.05.

To further clarify the role of *CgEAAT3* in the response of oysters to temperature stress, a detailed trend analysis of gene expression in oysters under high-temperature stress was conducted. A total of 5929 genes with similar expression trends to *CgEA*AT3 were identified (Figure 4F). The differential gene analysis of these similarly expressed genes under high temperature treatment revealed 963 DEGs with significant changes in both high temperature groups (Figure 4G). A KEGG enrichment analysis of the DEGs showed significant enrichment in eight metabolic pathways among the top 15 terms, with the top three being glutathione metabolism, taurine and hypotaurine metabolism, and arachidonic acid metabolism (Figure 4A). Additionally, ferroptosis and cell adhesion molecule-signaling pathways were significantly enriched, indicating that genes with similar trends to *CgEAAT3* under high-temperature stress are involved in the ferroptosis process.

To further explore the regulatory mechanism of *CgEAAT3* under high temperature, the expression patterns of *CgEAAT3* and *CgGPX4* under high temperature were examined. Results showed that *CgEAAT3* began to increase after 24 h of high temperature stimulation, peaking at 48 h (Figure 4B). *CgGPX4* also began to increase after 48 h, showing a similar trend to *CgEAAT3* (Figure 4C). Interfering with *CgEAAT3* expression under high-temperature stress led to the inhibition of *CgGPX4* (Figure 4D). Under high-temperature stress, the Fe content in the gills significantly increased, and it increased even further when *CgEAAT3* expression was interfered with (Figure 4E). This indicates that *CgEAAT3* can influence Fe transport in oyster gills by regulating *CgGPX4*.

## 3. Discussion

In this study, six glutamate transporter (GLT) genes were identified in the genome of the Pacific oyster (*Crassostrea gigas*), classified into the excitatory amino acid transporter (EAAT) and vesicular glutamate transporter (VGLUT) subfamilies. Through systematic analysis, these proteins exhibited unique structural characteristics and phylogenetic relationships. The *Cg*GLTs displayed diverse expression patterns at different developmental stages and in adult tissues, with their expression dynamics under high-temperature stress revealing specific regulatory mechanisms. Notably, *CgEAAT3* played a critical role in the response to high-temperature stress, potentially enhancing the oyster’s adaptability to environmental stress by regulating ferroptosis-related pathways. Our research deepens the understanding of the evolutionary roles of *Cg*GLTs, providing new theoretical foundations for elucidating their functional diversity and importance in stress responses.

During the larval development of *C. gigas*, the glutamate transporter family exhibits diverse expression patterns and functional characteristics. Studies indicate that GLTs regulate signal transmission between neurons, preventing neurotoxicity from excessive glutamate and ensuring proper neural development [18]. *Cg*GLTs peak in expression during the pediveliger to juvenile stages, when larvae develop eyespots and feet, increasing their environmental sensitivity and completing their attachment and metamorphosis [19]. In the early D-shaped larval stage, oyster larvae begin forming calcareous shells, with *CgVGLUT3* expression starting at this stage and gradually increasing with shell development. Glutamate is vital for maintaining bone cell homeostasis, and *VGLUT3* is necessary for glutamate release [20,21], suggesting that *CgVGLUT3* participates in shell formation. Interestingly, *CgEAATs* and *CgVGLUTs* exhibit complementary expression patterns during the blastula and early umbo stages, key periods for larval metamorphosis, reflecting different glutamate regulation mechanisms at various developmental stages. The expression patterns and functional characteristics of different glutamate transporters during oyster development highlight the crucial roles of glutamate in metabolism, environmental perception, and neural sensing, providing significant insights into oyster developmental biology and adaptive evolution.

The expression patterns of GLTs in adult Pacific oyster tissues closely correlate with the specific functions of each tissue. This study shows that *CgEAAT2*, *CgEAAT3*, and *CgVGLUT1* have higher expression in gills, labial palps, and the mantle. Gills and labial palps, essential for filter feeding and respiration, require high glutamate transport to support their metabolic activity and cellular communication [22,23]. The mantle, serving protective and secretory functions, also needs high levels of glutamate transport to maintain its roles. The rich neural networks in these tissues suggest that these genes may participate in the environmental perception processes of oysters [24,25]. *CgVGLUT3* is primarily expressed in hemocytes, critical immune cells in oysters, indicating its potential role in cellular immunity [26]. The high expression of *CgEAAT1* in gills and digestive glands highlights its role in nutrient absorption and waste excretion in these high-metabolic tissues. The high expression of GLTs at the inner and outer edges of the mantle further confirms their importance in oyster sensory systems and implies a role in shell formation. These findings reveal the critical roles of glutamate transporters in oyster environmental perception, immune defense, and shell formation, essential for understanding oyster physiology and adaptation.

The expression patterns of GLTs in *C. gigas* gills under high-temperature stress reveal distinct response characteristics. *CgEAAT1* and *CgVGLUT3* show similar expression trends, suggesting they may work together to maintain glutamate balance and stable neurotransmission during oyster adaptation to high temperatures. In contrast, *CgEAAT2* and *CgVGLUT1* show an initial increase and then a decrease from 20 °C to 30 °C, peaking at 25 °C, indicating a protective role at moderate temperatures but potential inhibition at higher temperatures. Notably, *CgEAAT3* and *CgVGLUT2* show opposite expression trends under high-temperature stress, reflecting different physiological regulatory mechanisms in oysters. *EAAT3* increases with rising temperatures, helping to clear excess glutamate from the synaptic cleft, preventing neurotoxicity, and improving glutamate reuse efficiency to maintain normal cell function [27]. Conversely, *VGLUT2* decreases, reflecting a protective response to reduce neural excitability and metabolic stress under high temperatures [28]. The changes in *Cg*GLT expression may enhance the temperature sensing ability of oysters, allowing better adaptation to environmental changes. The complex response patterns of *Cg*GLTs in *C. gigas* gills under high-temperature stress, especially the crucial role of *CgEAAT3*, highlight the key roles of *Cg*GLTs in oyster environmental stress response.

This study elucidates the pivotal role of *CgEAAT3* in the response to high-temperature stress in oysters, as revealed by gene expression analysis. Prior research has established that elevated temperatures induce oxidative stress, thereby impairing normal cellular and tissue functions [29,30]. Our findings demonstrate that high temperatures activate the glutathione metabolism pathway, enhancing the oxidative stress response. Notably, high-temperature stress significantly elevates the Fe content in the gills and enriches the ferroptosis pathway, indicating the regulation of ferroptosis in oyster gill cells under oxidative stress. GPX4 modulates lipid peroxide reduction and maintains antioxidant balance, protecting cells from ferroptosis and oxidative stress [10,31]. We observed that *CgEAAT3* and *CgGPX4* share similar expression patterns under high-temperature stress. Crucially, interference with *CgEAAT3* expression led to a marked downregulation of *CgGPX4* expression, accompanied by a further increase in the Fe content. These results suggest that *CgEAAT3* mediates ferroptosis in response to high-temperature stress through the regulation of *CgGPX4* expression. This study underscores the significant role of *CgEAAT3* in the high-temperature stress response of oysters, particularly in regulating ferroptosis and Fe metabolism. Our findings provide novel insights into the molecular mechanisms underlying oyster environmental stress responses and identify potential targets for enhancing oyster heat tolerance through genetic regulation.

The identification and classification of GLTs in various species reveal significant differences in gene diversity and function. Six GLTs were identified from the *C. gigas* genome, classified into the EAAT and VGLUT subfamilies. However, the GLT family in vertebrates (e.g., humans) is more diverse, comprising five EAATs (EAAT1-5), three VGLUTs (VGLUT1-3), and one glutamate–cysteine exchange transporter (SLC7A11/xCT) [2,7]. *EAAT4*, *EAAT5*, and *SLC7A11* were not found in oysters. However, *CgEAAT1-3* and *CgVGLUT1-3* show evolutionary conservation across different species, indicating their fundamental and essential roles in neurotransmission and cellular protection. The complex nervous system of vertebrates can quickly respond to environmental information. For example, EAAT4 and EAAT5 regulate glutamate concentration in the cerebellum and retina, respectively [32,33]. In contrast, the simpler nervous systems of invertebrates, like oysters, do not require such diverse glutamate transport mechanisms, resulting in less gene amplification. The complexity of vertebrate environments and the demands of their nervous systems may drive the amplification and diversification of GLTs. Gene amplification provides the basis for functional differentiation, allowing GLTs to perform specific roles in different tissues and cell types, better adapting to complex physiological needs and environmental challenges.

Glutamate transporters are crucial for regulating glutamate uptake and release in the nervous system [18]. *Cg*EAATs and *Cg*VGLUTs in *C. gigas* exhibit distinct structural characteristics and show evolutionary conservation. The GltP domain in *Cg*EAATs primarily mediates the proton-coupled transport of glutamate and aspartate. It shows high homology with EAATs from humans and other species, emphasizing its essential role in oyster neurotransmission [34]. The MFS domain in *Cg*VGLUTs, as the largest family of secondary carriers, is involved in transporting various substrates. Through evolutionary duplication, this domain generates multiple functional proteins, likely participating in the transmembrane transport of nutrients and metabolites in oysters, enhancing their adaptability to complex marine environments [35]. The high conservation of these proteins aids in understanding oyster neurotransmission mechanisms and underscores their importance in adaptive evolution. VGLUTs store the excitatory neurotransmitter Glu in synaptic vesicles, while EAATs clear it from the synaptic cleft to maintain low extracellular concentrations and prevent neurotoxicity [36]. The conservation of *Cg*EAATs and *Cg*VGLUTs likely enhances the survival capability of *C. gigas* in complex marine environments by ensuring efficient neurotransmission and neurotransmitter balance.

## 4. Materials and Methods

### 4.1. Identification and Functional Annotation of the Glutamate Transporter Family Genes in C. gigas

To identify glutamate transporter family genes in *C. gigas*, we searched the transcriptome and genome sequences using amino acid sequences of vertebrate and invertebrate glutamate transporters from the NCBI (https://www.ncbi.nlm.nih.gov/, accessed on 1 December 2022) and Uniprot (https://www.uniprot.org/, accessed on 1 December 2022) databases. Candidate genes were validated through phylogenetic analysis. Conserved domains within the glutamate transporter gene family were identified using the NCBI CDD program, while conserved motifs were analyzed via the MEME online tool (https://meme-suite.org/meme/tools/meme, accessed on 25 December 2022), with a maximum of 10 motifs. The molecular weight and isoelectric points of the glutamate transporter family members were determined using the ExPASy tool (https://web.expasy.org/protparam/, accessed on 20 January 2023). Protein structures of the identified glutamate transporters were visualized using IBS1.0.3 software [37], and TBtools (V2.101) was used for further visualization [38].

### 4.2. Chromosomal Distribution and Phylogenetic Analysis of the Glutamate Transporter Family in C. gigas

The physical locations of the glutamate transporter genes on the *C. gigas* chromosomes were extracted from the NCBI *C. gigas* genome database. The glutamate transporters were mapped to five chromosomes. A phylogenetic tree was constructed using glutamate transporter sequences from 17 species (Supplementary Table S1), obtained from the NCBI and Uniprot databases. The neighbor-joining method with 1000 bootstrap replicates in MEGA-X was used for phylogenetic analysis.

### 4.3. Spatiotemporal Expression Profiling

The RPKM (reads per kilobase per million mapped reads) values for different developmental stages and tissue distributions of the glutamate transporter genes were obtained from the published *C. gigas* RNA-seq dataset (PRJNA146329). Radar charts were created using the R package to display expression changes at different developmental stages. A heatmap of tissue distribution was created by log_2_-transforming the RPKM values using TBtools.

### 4.4. CgEAAT3 Pathway Enrichment and Network Analysis

Following Venn diagram analysis, KEGG enrichment analysis was conducted on all intersecting data using the R package. Pathways involving the *CgEAAT3* gene were identified, and network maps of these pathways were generated using OmicShare tools. Detailed information on the enriched pathways and associated genes is provided in Supplementary Table S2.

### 4.5. Experimental Materials and High-Temperature Stress Treatment

Adult Pacific oysters (*Crassostrea gigas*, 2–3 years old) were collected from Changhai County, Dalian, Liaoning Province. The high-temperature stress was conducted at 28 °C to simulate typical summer surface water temperatures in the shellfish breeding areas of the northern Yellow Sea. Before experimentation, the oysters were acclimated for 7 days in aerated, filtered seawater maintained at 15 ± 2 °C, with the water changed every 48 h. They were fed daily with commercial spirulina powder pre-filtered through a 300-mesh screen. After acclimation, the oysters were exposed to 28 °C for 0, 6, 24, 48, and 72 h. At each time point, gill tissues were dissected, immediately frozen in liquid nitrogen, and stored at −80 °C for subsequent RNA extraction.

For RNA interference experiments, dsRNA expression vectors were constructed and introduced into RNase III-deficient *Escherichia coli* HT115 (DE3). The dsRNA fragments of *CgEAAT3* and *EGFP* were induced by IPTG. Eighteen oysters were divided into three groups: a blank group, an experimental group (*CgEAAT3*-RNAi), and a control group (*EGFP*-RNAi). The experimental group and the control group were injected with 100 μL of 1000 ng/μL *CgEAAT3* and *EGFP* dsRNA fragments into the adductor muscle of oysters. Twelve hours after the first injection, a second dose was administered, and oysters were transferred to a 28 °C tank. After 48 h, gill tissues were collected for the qRT-PCR analysis of *CgEAAT3* and *CgGPX4* expression, with the total iron content measured using the Tissue Iron Assay Kit (Nanjing Jiancheng Bioengineering Institute, Nanjing, China). Gill tissues from two oysters were pooled into one sample, with three replicates per group.

### 4.6. Expression of Glutamate Transporters in C. Gigas Under High-Temperature Stress

Using the RNA-Seq dataset (PRJNA146329) from the NCBI database, the expression patterns of glutamate transporters in *C. gigas* gills were analyzed after 7 days of exposure to different temperatures (20 °C, 25 °C, 30 °C) [39]. The data were mapped to the *C. gigas* genome using HISAT2 (v2.0.5) with default settings, and gene expression levels were estimated using the RPKM method. Log_2_-transformed RPKM values were used to generate a heatmap via TBtools. Differential gene expression analysis across temperature conditions was conducted using the online platform Omicshare (https://www.omicshare.com/tools/, accessed on 1 April 2023), and a Venn diagram was constructed. Time-series trend analysis and gene expression clustering under temperature stress were performed using the Mfuzz R package (version 4.2.3) [40].

### 4.7. Total RNA Extraction and qRT-PCR

Total RNA was extracted from the gill tissues of *C. gigas* using the TRIzol method. The RNA quality was checked using NanoDrop 2000 (Thermo Fisher Scientific, Waltham, MA, USA) and nucleic acid gel electrophoresis. According to the protocol provided by the manufacturer, reverse transcription was performed using the One-Step gDNA Removal and cDNA Synthesis SuperMix kit (TransScript, Beijing, China). Primers were designed using Primer Premier 5 software, with the *C. gigas* EF gene as the internal reference gene. Three gill tissue samples were used for each time point, with three technical replicates per sample. Real-time quantitative PCR was performed using NovoStart^®^SYBR qPCR SuperMix Plus (Novoprotein, Suzhou, China), and gene expression levels were calculated using the comparative cycle threshold (2^−∆∆CT^) method. All the primers are listed in Table 3.

### 4.8. Data Analysis

All data were calculated as mean ± standard deviation and analyzed for significance using SPSS 27 software. Statistical significance was defined as *p* < 0.05, and highly significant differences were defined as *p* < 0.01 (*n* = 3). All experiments were performed in accordance with the approval and guidelines of the Ethics Review Committee of Dalian Ocean University.

## 5. Conclusions

In summary, this study systematically analyzed the glutamate transporter (GLT) family in the *C. gigas*, focusing on their expression and regulatory mechanisms under high-temperature stress. We identified six GLT genes, categorized them into two subfamilies, and elucidated their expression patterns across different developmental stages and tissues. Notably, *CgEAAT3* was significantly upregulated under high-temperature stress, indicating its sensitivity and broad response to temperature fluctuations. KEGG enrichment analysis revealed that high-temperature stress significantly affects the glutathione metabolism and ferroptosis pathways. Further investigation showed that under elevated temperatures, *CgEAAT3* plays a critical role in modulating ferroptosis and maintaining redox homeostasis by regulating *CgGPX4* expression. This study offers new insights into GLT evolution and stress responses in mollusks, providing a theoretical foundation for understanding potential adaptations in marine bivalves under global warming.

## Figures and Tables

**Table 1:** Sequence characteristics of glutamate transporter gene family of *C. gigas*.

Gene Name	Gene ID	cDNA Length (bp)	ORF Length (bp)	Exons No.	Introns No.	Amino Acid No.	Molecular Weight (kDa)	Theoretical PI	AlpHa No.	Beta No.	Colins No.	Turn No.	GRAVY of PD
*EAAT1*	LOC105330890	2298	1563	10	9	520	56.16	6.97	21	31	33	27	0.500
*EAAT2*	LOC105335486	3700	1713	10	9	570	62.80	5.30	30	38	36	32	0.342
*EAAT3*	LOC105332706	2720	1707	8	7	568	61.46	5.25	26	31	29	28	0.548
*VGLUT1*	LOC105331066	3556	1887	12	11	628	69.61	6.17	21	40	50	50	0.129
*VGLUT2*	LOC105342017	2003	1689	13	12	562	62.50	6.02	23	42	44	51	0.145
*VGLUT3*	LOC105324413	1962	1467	13	12	488	53.14	8.87	23	34	32	35	0.564

**Table 2:** Percentage of *C. gigas* glutamate transporters with selected GLTs proteins in other species.

Gene	*H. sapiens*	*M. musculus*	*D. rerio*	*X. tropicalis*	*G. gallus*	*S. pistillata*	*D. magna*	*S. purpuratus*	*P. vulgata*
*EAAT1*	53.80%	52.58%	53.70%	50.10%	53.65%	40.15%	50.1%	47.66%	52.04%
*EAAT2*	55.22%	52.11%	55.34%	56.34%	54.9%	41.29%	/	51.73%	60.18%
*EAAT3*	46.73%	49.50%	44.87%	48.76%	48.16%	47.65%	47.02%	58.7%	/
*VGLUT1*	60.63%	60.63%	/	51.63%	/	35.53%	56.43%	58.24%	39.49%
*VGLUT2*	33.33%	33.60%	32.44%	32.92%	33.88%	33.65%	33.61%	/	33.41%
*VGLUT3*	34.58%	35.32%	36.78%	32.99%	34.24%	/	/	/	/

**Table 3:** Sequences of the primers used in the present study.

Primer Name	Sequence (5′–3′)	Product Length (bp)	Application
*CgEAAT3*-RT-F	AATTGGCGAGAAAGGAAGG	120	RT-PCR
*CgEAAT3*-RT-R	GCGGCGATAAGAAAGGCT		
*CgGPX4*-RT-F	AAAGTATGCTGAGGAGAAGGGGCT	273	RT-PCR
*CgGPX4*-RT-R	CTTTTCACTGGCTTCCCTTCTTTG		
*CgEF*-RT-F	AGTCACCAAGGCTGCACAGAAAG	201	RT-PCR
*CgEF*-RT-R	TCCGACGTATTTCTTTGCGATGT		
*CgEAAT3*-Fi	CCCAAGCTTATGACAACAGTTGCACCCAA	498	RNA interference
*CgEAAT3*-Ri	CTAGCTAGCGTCCACGTTGTTTCGTTCCT		
*EGFP*-F	CCCAAGCTTACGTAAACGGCCACAAGTTC	495	RNA interference
*EGFP*-R	CTAGCTAGCTGTTCTGCTGGTAGTGGTCG		

## Data Availability

The original contributions presented in this study are included in the article/Supplementary Materials; further inquiries can be directed to the corresponding authors.

## Supplementary Materials

The following supporting information can be downloaded at https://www.mdpi.com/article/10.3390/ijms252111342/s1.
