# Supplementary material for: Regulation Mechanisms of the Glutamate Transporter in the Response of Pacific Oyster upon High-Temperature Stress
Source: Int J Mol Sci. 2024 Oct 22;25(21):11342. doi: 10.3390/ijms252111342 (PMC11545548; doi:10.3390/ijms252111342)
Supplement: Supplementary file 1 [file ijms-25-11342-s001.zip › ijms-3246609-supplementary.pdf]

Table S1 Statistical information on the amino acid sequences of the Glutamate transporters of the selected species (search databases: NCBI, Uniprot).

Species Name	Abbreviation	Latin name	Protein ID (Uniprot/NCBI)	Gene Name	Application
Human	H.SA	*Homo sapiens*	NP_004163.3	*EAAT1*	Phylogenetic analysis; Identification
Human	H.SA	*Homo sapiens*	NP_004162.2	*EAAT2*	Phylogenetic analysis; Identification
Human	H.SA	*Homo sapiens*	NP_004161.4	*EAAT3*	Phylogenetic analysis; Identification
Human	H.SA	*Homo sapiens*	NP_064705.1	*VGLUT1*	Phylogenetic analysis; Identification
Human	H.SA	*Homo sapiens*	NP_065079.1	*VGLUT2*	Phylogenetic analysis; Identification
Human	H.SA	*Homo sapiens*	NP_647480.1	*VGLUT3*	Phylogenetic analysis; Identification
House mouse	M.MU	*Mus musculus*	NP_683740.1	*EAAT1*	Phylogenetic analysis; Identification
House mouse	M.MU	*Mus musculus*	NP_001070982.1	*EAAT2*	Phylogenetic analysis; Identification
House mouse	M.MU	*Mus musculus*	NP_033225.1	*EAAT3*	Phylogenetic analysis; Identification
House mouse	M.MU	*Mus musculus*	NP_892038.2	*VGLUT1*	Phylogenetic analysis; Identification
House mouse	M.MU	*Mus musculus*	Q8BLE7.1	*VGLUT2*	Phylogenetic analysis; Identification
House mouse	M.MU	*Mus musculus*	NP_892004.1	*VGLUT3*	Phylogenetic analysis; Identification
Pigeons	C.LI	*Columba livia*	XP_005508073.1	*EAAT1*	Phylogenetic analysis
Pigeons	C.LI	*Columba livia*	NP_001269759.1	*EAAT2*	Phylogenetic analysis
Pigeons	C.LI	*Columba livia*	XP_021149763.1	*EAAT3*	Phylogenetic analysis
Pigeons	C.LI	*Columba livia*	NP_001269762.1	*VGLUT2*	Phylogenetic analysis
Pigeons	C.LI	*Columba livia*	AIT55235.1	*VGLUT3*	Phylogenetic analysis
Frog	X.TR	*Xenopus tropicalis*	NP_001106687.1	*EAAT1*	Phylogenetic analysis; Identification
Frog	X.TR	*Xenopus tropicalis*	XP_017951228.2	*EAAT2*	Phylogenetic analysis; Identification
Frog	X.TR	*Xenopus tropicalis*	XP_031746513.1	*EAAT3*	Phylogenetic analysis; Identification

Frog	X.TR	*Xenopus tropicalis*	NP_001072608.1	*VGLUT1*	Phylogenetic analysis; Identification
Frog	X.TR	*Xenopus tropicalis*	XP_031756249.1	*VGLUT2*	Phylogenetic analysis; Identification
Frog	X.TR	*Xenopus tropicalis*	XP_002936892.1	*VGLUT3*	Phylogenetic analysis; Identification
Zebrafish	D.RE	*Danio rerio*	XP_009299445.1	*EAAT1*	Phylogenetic analysis; Identification
Zebrafish	D.RE	*Danio rerio*	XP_009296497.1	*EAAT2*	Phylogenetic analysis; Identification
Zebrafish	D.RE	*Danio rerio*	NP_001002666.2	*EAAT3*	Phylogenetic analysis; Identification
Zebrafish	D.RE	*Danio rerio*	XP_009295917.2	*VGLUT1-like*	Phylogenetic analysis; Identification
Zebrafish	D.RE	*Danio rerio*	NP_001009982.1	*VGLUT2*	Phylogenetic analysis; Identification
Zebrafish	D.RE	*Danio rerio*	NP_001076304.1	*VGLUT3*	Phylogenetic analysis; Identification
Chicken	G.GA	*Gallus gallus*	XP_046791432.1	*EAAT1*	Identification
Chicken	G.GA	*Gallus gallus*	NP_001384072.1	*EAAT2*	Identification
Chicken	G.GA	*Gallus gallus*	XP_046792511.1	*EAAT3*	Identification
Chicken	G.GA	*Gallus gallus*	NP_001161855.2	*VGLUT2*	Identification
Chicken	G.GA	*Gallus gallus*	NP_001384126.1	*VGLUT3*	Identification
Water flea	D.MA	*Daphnia magna*	XP_045026376.1	*EAAT1*	Phylogenetic analysis; Identification
Water flea	D.MA	*Daphnia magna*	XP_032780850.2	*EAAT3*	Phylogenetic analysis; Identification
Water flea	D.MA	*Daphnia magna*	XP_045031560.1	*VGLUT1*	Phylogenetic analysis; Identification
Water flea	D.MA	*Daphnia magna*	KZS04443.1	*VGLUT2*	Phylogenetic analysis; Identification
Purple sea urchin	S.PU	*Strongylocentrotus purpuratus*	XP_030852710.1	*EAAT1*	Phylogenetic analysis; Identification
Purple sea urchin	S.PU	*Strongylocentrotus purpuratus*	XP_030845491.1	*EAAT2*	Phylogenetic analysis; Identification
Purple sea urchin	S.PU	*Strongylocentrotus purpuratus*	XP_781833.4	*EAAT3*	Phylogenetic analysis; Identification
Purple sea urchin	S.PU	*Strongylocentrotus purpuratus*	XP_030845556.1	*VGLUT1*	Phylogenetic analysis; Identification
Purple sea urchin	S.PU	*Strongylocentrotus purpuratus*	XP_030843286.1	*VGLUT3-like*	Phylogenetic analysis; Identification

Lingula	L.AN	*Lingula anatina*	XP_013416271.1	*EAAT1*	Phylogenetic analysis
Lingula	L.AN	*Lingula anatina*	XP_013388207.1	*EAAT2-like*	Phylogenetic analysis
Lingula	L.AN	*Lingula anatina*	XP_023933109.1	*EAAT3*	Phylogenetic analysis
Lingula	L.AN	*Lingula anatina*	XP_013399801.1	*VGLUT1*	Phylogenetic analysis
Lingula	L.AN	*Lingula anatina*	XP_013404116.1	*VGLUT2*	Phylogenetic analysis
Lingula	L.AN	*Lingula anatina*	XP_013392278.1	*VGLUT3*	Phylogenetic analysis
Sea cucumber	H.LE	*Holothuria leucospilota*	KAJ8040715.1	*EAAT1*	Phylogenetic analysis
Sea cucumber	H.LE	*Holothuria leucospilota*	KAJ8031400.1	*EAAT2*	Phylogenetic analysis
Sea cucumber	H.LE	*Holothuria leucospilota*	KAJ8040326.1	*EAAT3*	Phylogenetic analysis
Sea cucumber	H.LE	*Holothuria leucospilota*	KAJ8023897.1	*VGLUT2*	Phylogenetic analysis
Sea cucumber	H.LE	*Holothuria leucospilota*	KAJ8037927.1	*VGLUT3*	Phylogenetic analysis
Smooth cauliflower coral	S.PI	*Stylophora pistillata*	PFX32517.1	*EAAT1*	Phylogenetic analysis; Identification
Smooth cauliflower coral	S.PI	*Stylophora pistillata*	PFX32520.1	*EAAT2*	Phylogenetic analysis; Identification
Smooth cauliflower coral	S.PI	*Stylophora pistillata*	PFX32505.1	*EAAT3*	Phylogenetic analysis; Identification
Smooth cauliflower coral	S.PI	*Stylophora pistillata*	PFX25698.1	*VGLUT1*	Phylogenetic analysis; Identification
Smooth cauliflower coral	S.PI	*Stylophora pistillata*	PFX25882.1	*VGLUT2*	Phylogenetic analysis; Identification
Smooth cauliflower coral	S.PI	*Stylophora pistillata*	XP_022797010.1	*VGLUT3-like*	Phylogenetic analysis; Identification
Oriental fruit fly	B.DO	*Bactrocera dorsalis*	XP_049306780.1	*EAAT1*	Phylogenetic analysis
Oriental fruit fly	B.DO	*Bactrocera dorsalis*	JAC37776.1	*EAAT2*	Phylogenetic analysis
Oriental fruit fly	B.DO	*Bactrocera dorsalis*	XP_049311798.1	*VGLUT1*	Phylogenetic analysis
Oriental fruit fly	B.DO	*Bactrocera dorsalis*	JAC42875.1	*VGLUT2*	Phylogenetic analysis
Oriental fruit fly	B.DO	*Bactrocera dorsalis*	JAC48577.1	*VGLUT3*	Phylogenetic analysis
Yesso scallop	M.YE	*Mizuhopecten yessoensis*	OWF39054.1	*EAAT1*	Phylogenetic analysis
Yesso scallop	M.YE	*Mizuhopecten yessoensis*	OWF45538.1	*EAAT2*	Phylogenetic analysis
Yesso scallop	M.YE	*Mizuhopecten yessoensis*	OWF44783.1	*EAAT3*	Phylogenetic analysis
Yesso scallop	M.YE	*Mizuhopecten yessoensis*	XP_021340318.1	*VGLUT1-like*	Phylogenetic analysis

Yesso scallop	M.YE	*Mizuhopecten yessoensis*	OWF36573.1	*VGLUT2*	Phylogenetic analysis
Portuguese oyster	C.AN	*Crassostrea angulata*	XP_052690074.1	*EAAT1-like*	Phylogenetic analysis
Portuguese oyster	C.AN	*Crassostrea angulata*	XP_052704024.1	*EAAT2-like*	Phylogenetic analysis
Portuguese oyster	C.AN	*Crassostrea angulata*	XP_052704024.1	*EAAT3-like*	Phylogenetic analysis
Portuguese oyster	C.AN	*Crassostrea angulata*	XP_052715853.1	*VGLUT1-like*	Phylogenetic analysis
Portuguese oyster	C.AN	*Crassostrea angulata*	XP_052688672.1	*VGLUT2-like*	Phylogenetic analysis
Portuguese oyster	C.AN	*Crassostrea angulata*	XP_052699653.1	*VGLUT3-like*	Phylogenetic analysis
European flat oyster	O.ED	*Ostrea edulis*	XP_048755442.2	*EAAT1-like*	Phylogenetic analysis
European flat oyster	O.ED	*Ostrea edulis*	XP_048742341.2	*EAAT2-like*	Phylogenetic analysis
European flat oyster	O.ED	*Ostrea edulis*	XP_056007353.1	*EAAT3-like*	Phylogenetic analysis
European flat oyster	O.ED	*Ostrea edulis*	XP_048734554.2	*VGLUT1-like*	Phylogenetic analysis
European flat oyster	O.ED	*Ostrea edulis*	XP_048780550.2	*VGLUT2-like*	Phylogenetic analysis
European flat oyster	O.ED	*Ostrea edulis*	XP_048780550.2	*VGLUT3-like*	Phylogenetic analysis
Limpets	P.VU	*Patella vulgata*	XP_050388904.1	*EAAT1*	Identification
Limpets	P.VU	*Patella vulgata*	XP_050391421.1	*EAAT2*	Identification
Limpets	P.VU	*Patella vulgata*	XP_050416582.1	*VGLUT1*	Identification
Limpets	P.VU	*Patella vulgata*	XP_050409086.2	*VGLUT2*	Identification
Eastern oyster	C.VI	*Crassostrea virginica*	XP_022318595.1	*EAAT1-like*	Phylogenetic analysis
Eastern oyster	C.VI	*Crassostrea virginica*	XP_022335624.1	*EAAT2-like*	Phylogenetic analysis
Eastern oyster	C.VI	*Crassostrea virginica*	XP_022309875.1	*EAAT3-like*	Phylogenetic analysis
Eastern oyster	C.VI	*Crassostrea virginica*	XP_022325986.1	*VGLUT1-like*	Phylogenetic analysis
Eastern oyster	C.VI	*Crassostrea virginica*	XP_022341234.1	*VGLUT2-like*	Phylogenetic analysis
Eastern oyster	C.VI	*Crassostrea virginica*	XP_022333927.1	*VGLUT3-like*	Phylogenetic analysis
Blacklip abalone	H.RU	*Haliotis rubra*	XP_046551544.1	*EAAT1-like*	Phylogenetic analysis
Blacklip abalone	H.RU	*Haliotis rubra*	XP_046548237.1	*EAAT2-like*	Phylogenetic analysis
Blacklip abalone	H.RU	*Haliotis rubra*	XP_046580436.1	*EAAT3-like*	Phylogenetic analysis
Blacklip abalone	H.RU	*Haliotis rubra*	XP_046546501.1	*VGLUT1-like*	Phylogenetic analysis
Blacklip abalone	H.RU	*Haliotis rubra*	XP_046546501.1	*VGLUT2-like*	Phylogenetic analysis
Blacklip abalone	H.RU	*Haliotis rubra*	XP_046549718.1	*VGLUT3-like*	Phylogenetic analysis
Pacific oyster	C.GI	*Crassostrea gigas*	XP_011431127.2	*EAAT1*	Phylogenetic analysis
Pacific oyster	C.GI	*Crassostrea gigas*	XP_011437656.2	*EAAT2*	Phylogenetic analysis

Pacific oyster	C.GI	*Crassostrea gigas*	XP_011433693.2	*EAAT3*	Phylogenetic analysis
Pacific oyster	C.GI	*Crassostrea gigas*	XP_011431416.2	*VGLUT1*	Phylogenetic analysis
Pacific oyster	C.GI	*Crassostrea gigas*	XP_011447099.2	*VGLUT2*	Phylogenetic analysis
Pacific oyster	C.GI	*Crassostrea gigas*	XP_011421779.2	*VGLUT3*	Phylogenetic analysis

Table S2 Statistical table of KEGG-enriched gene.

KEGG_A_ class	KEGG_B_ class	Pathway	out (56)	All (13488)	Pvalue	Qvalue	Pathway ID	Genes	K_IDs
Metabolism	Metabolism of other amino acids	Taurine and hypotaurine metabolism	8	52	3.43E-11	1.77E-09	ko00430	XP_011420516.2; XP_011420517.2; XP_019920816.2; XP_034318992.1; XP_034319010.1; XP_034319018.1; XP_034319027.1; XP_034319035.1	K18592+ K18592+ K18592+ K18592+ K18592+ K18592+ K18592+ K18592
Metabolism	Metabolism of other amino acids	Glutathione metabolism	10	120	5.36E-11	1.77E-09	ko00480	XP_011420516.2; XP_011420517.2; XP_011425776.2; XP_011425777.2; XP_019920816.2; XP_034318992.1; XP_034319010.1; XP_034319018.1; XP_034319027.1; XP_034319035.1	K18592+ K18592+ K00682+ K00682+ K18592+ K18592+ K18592+ K18592+ K18592+ K18592
Metabolism	Lipid metabolism	Arachidonic acid metabolism	8	150	1.75E-07	3.86E-06	ko00590	XP_011420516.2; XP_011420517.2; XP_019920816.2; XP_034318992.1; XP_034319010.1; XP_034319018.1; XP_034319027.1; XP_034319035.1	K18592+ K18592+ K18592+ K18592+ K18592+ K18592+ K18592+ K18592
Metabolism	Biosynthesis of other secondary metabolites	Penicillin and cephalosporin biosynthesis	2	4	0.000101046	1.67E-03	ko00311	XP_011419076.2; XP_019920382.2	K00273+ K00273
Genetic Information Processing	Translation	Aminoacyl-tRNA biosynthesis	4	82	0.000366472	4.84E-03	ko00970	XP_019929472.2; XP_034314410.1; XP_034337579.1; XP_034337580.1	K01881+ K01881+ K01887+ K01887

Metabolism	Global and overview maps	Metabolic pathways	24	3315	0.0020169	2.22E-02	ko01100	XP_011419076.2; XP_011420126.2; XP_011420516.2; XP_011420517.2; XP_011420577.2; XP_011425776.2; XP_011425777.2; XP_011432861.1; XP_011447576.2; XP_011450602.3; XP_011454785.1; XP_019920382.2; XP_019920816.2; XP_034299460.1; XP_034300155.1; XP_034309873.1; XP_034318992.1; XP_034319010.1; XP_034319018.1; XP_034319027.1; XP_034319035.1; XP_034324622.1; XP_034336035.1; XP_034336409.1	K00273+ K20796+ K18592+ K18592+ K20247+ K00682+ K00682+ K05287+ K10249+ K03859+ K01519+ K00273+ K18592+ K20796+ K14073+ K13566+ K18592+ K18592+ K18592+ K18592+ K18592+ K00965+ K01229+ K12323
Metabolism	Metabolism of other amino acids	D-Amino acid metabolism	2	24	0.004406899	3.80E-02	ko00470	XP_011419076.2; XP_019920382.2	K00273+ K00273
Genetic Information Processing	Translation	Ribosome biogenesis in eukaryotes	3	81	0.004600416	3.80E-02	ko03008	XP_011453742.2; XP_034304630.1; XP_034304631.1	K11108+ K03539+ K03539
Human Diseases	Drug resistance: antineoplastic	Antifolate resistance	3	97	0.007587595	5.12E-02	ko01523	XP_034320754.1; XP_034320755.1; XP_034320756.1	K05667+ K05667+ K05665
Metabolism	Glycan biosynthesis and metabolism	Glycosylphosphatidylinositol (GPI)-anchor biosynthesis	2	32	0.007753284	5.12E-02	ko00563	XP_011432861.1; XP_011450602.3	K05287+ K03859
Genetic Information Processing	Replication and repair	Fanconi anemia pathway	3	106	0.009671494	5.80E-02	ko03460	XP_019926742.2; XP_034338515.1; XP_034338516.1	K10891+ K10892+ K10892
Environmental Information Processing	Membrane transport	ABC transporters	3	113	0.01150139	6.33E-02	ko02010	XP_034320754.1; XP_034320755.1; XP_034320756.1	K05667+ K05667+ K05665
Cellular Processes	Cell growth and death	Ferroptosis	2	56	0.022594	1.15E-01	ko04216	NP_001292237.1; XP_011444077.2	K21398+ K21398
Metabolism	Carbohydrate metabolism	Galactose metabolism	2	62	0.02730936	1.29E-01	ko00052	XP_034324622.1; XP_034336035.1	K00965+ K01229
Environmental Information Processing	Signaling molecules and interaction	Cell adhesion molecules	3	185	0.04142851	1.76E-01	ko04514	XP_011433258.2; XP_011442801.2; XP_034316267.1	K05693+ K07378+ K05693
Metabolism	Amino acid metabolism	Glycine, serine and threonine metabolism	2	79	0.04255627	1.76E-01	ko00260	XP_011419076.2; XP_019920382.2	K00273+ K00273
Environmental Information Processing	Signal transduction	TGF-beta signaling pathway	2	105	0.07048028	2.74E-01	ko04350	XP_011420182.1; XP_034311867.1	K04666+ K04666
Metabolism	Amino acid metabolism	Lysine degradation	2	122	0.09117976	3.26E-01	ko00310	XP_011420126.2; XP_034299460.1	K20796+ K20796
Organismal Systems	Digestive system	Vitamin digestion and absorption	2	125	0.09500065	3.26E-01	ko04977	XP_034300155.1; XP_034320756.1	K14073+ K05665
Environmental Information Processing	Signaling molecules and interaction	Cytokine-cytokine receptor interaction	2	128	0.09886747	3.26E-01	ko04060	XP_011420182.1; XP_034311867.1	K04666+ K04666

Metabolism	Amino acid metabolism	Arginine and proline metabolism	2	140	0.114761 1	3.44E-01	ko00 330	XP_011419076.2; XP_019920382.2	K00273+ K00273
Organismal Systems	Digestive system	Mineral absorption	2	140	0.114761 1	3.44E-01	ko04 978	NP_001292237.1; XP_011444077.2	K21398+ K21398
Organismal Systems	Digestive system	Protein digestion and absorption	2	167	0.152596 9	4.32E-01	ko04 974	XP_011442773.2; XP_034307100.1	K14208+ K01298
Cellular Processes	Transport and catabolism	Peroxisome	2	170	0.156945 2	4.32E-01	ko04 146	XP_011419076.2; XP_019920382.2	K00273+ K00273
Organismal Systems	Digestive system	Bile secretion	2	192	0.189489	5.00E-01	ko04 976	XP_034320754.1; XP_034320755.1	K05667+ K05667
Metabolism	Amino acid metabolism	Histidine metabolism	1	53	0.198229 3	5.01E-01	ko00 340	XP_011420577.2	K20247
Metabolism	Lipid metabolism	Fatty acid elongation	1	55	0.204899 5	5.01E-01	ko00 062	XP_011447576.2	K10249
Cellular Processes	Cellular community - eukaryotes	Signaling pathways regulating pluripotency of stem cells	2	214	0.222876	5.25E-01	ko04 550	XP_011420182.1; XP_034311867.1	K04666+ K04666
Organismal Systems	Digestive system	Carbohydrate digestion and absorption	1	70	0.253218 6	5.49E-01	ko04 973	XP_034336035.1	K01229
Metabolism	Lipid metabolism	Biosynthesis of unsaturated fatty acids	1	72	0.259439 2	5.49E-01	ko01 040	XP_011447576.2	K10249
Organismal Systems	Nervous system	Synaptic vesicle cycle	2	241	0.264437 5	5.49E-01	ko04 721	XP_011433693.2; XP_011445052.2	K05617+ K05613
Organismal Systems	Nervous system	Glutamatergic synapse	2	242	0.265981 8	5.49E-01	ko04 724	XP_011433693.2; XP_011445052.2	K05617+ K05613
Organismal Systems	Endocrine system	Prolactin signaling pathway	1	89	0.3103	5.75E-01	ko04 917	XP_034324622.1	K00965
Organismal Systems	Digestive system	Fat digestion and absorption	1	89	0.3103	5.75E-01	ko04 975	XP_034300155.1	K14073
Metabolism	Amino acid metabolism	Alanine, aspartate and glutamate metabolism	1	90	0.313182 6	5.75E-01	ko00 250	XP_034309873.1	K13566
Cellular Processes	Cellular community - eukaryotes	Adherens junction	2	274	0.315309 4	5.75E-01	ko04 520	XP_011433258.2; XP_034316267.1	K05693+ K05693
Organismal Systems	Endocrine system	Oxytocin signaling pathway	3	481	0.322453 9	5.75E-01	ko04 921	XP_034319164.1; XP_034319165.1; XP_034336409.1	K04977+ K04977+ K12323
Organismal Systems	Endocrine system	Regulation of lipolysis in adipocytes	1	125	0.406946 5	6.78E-01	ko04 923	XP_034336409.1	K12323
Metabolism	Global and overview maps	Fatty acid metabolism	1	131	0.421705 6	6.78E-01	ko01 212	XP_011447576.2	K10249
Genetic Information Processing	Folding, sorting and degradation	RNA degradation	1	139	0.440824 6	6.78E-01	ko03 018	XP_034330911.1	K03654
Organismal Systems	Digestive system	Pancreatic secretion	2	364	0.448610 1	6.78E-01	ko04 972	XP_034300155.1; XP_034307100.1	K14073+ K01298
Organismal Systems	Immune system	NOD-like receptor signaling pathway	2	365	0.450018 1	6.78E-01	ko04 621	XP_034319164.1; XP_034319165.1	K04977+ K04977
Metabolism	Lipid metabolism	Glycerolipid metabolism	1	143	0.450149 8	6.78E-01	ko00 561	XP_034300155.1	K14073
Metabolism	Xenobiotics biodegradat ion and metabolism	Drug metabolism - other enzymes	1	146	0.457043 4	6.78E-01	ko00 983	XP_011454785.1	K01519
Metabolism	Nucleotide metabolism	Purine metabolism	2	374	0.462602	6.78E-01	ko00 230	XP_011454785.1; XP_034336409.1	K01519+ K12323

Cellular Processes	Transport and catabolism	Lysosome	2	409	0.5099451	7.32E-01	ko04142	NP_001292237.1; XP_011444077.2	K21398+K21398
Human Diseases	Infectious disease: bacterial	Epithelial cell signaling in Helicobacter pylori infection	1	182	0.533429	7.49E-01	ko05120	XP_034318741.1	K08114
Metabolism	Carbohydrate metabolism	Amino sugar and nucleotide sugar metabolism	1	214	0.5923976	8.13E-01	ko00520	XP_034324622.1	K00965
Human Diseases	Neurodegenerative disease	Parkinson disease	2	485	0.6032199	8.13E-01	ko05012	NP_001292237.1; XP_011444077.2	K21398+K21398
Environmental Information Processing	Signal transduction	Sphingolipid signaling pathway	1	247	0.6455351	8.51E-01	ko04071	XP_034320756.1	K05665
Human Diseases	Neurodegenerative disease	Spinocerebellar ataxia	1	255	0.6573552	8.51E-01	ko05017	XP_011433693.2	K05617
Organismal Systems	Endocrine system	Aldosterone synthesis and secretion	1	286	0.6996107	8.88E-01	ko04925	XP_034336409.1	K12323
Cellular Processes	Transport and catabolism	Phagosome	1	319	0.7389709	9.13E-01	ko04145	XP_019928009.2	K06560
Organismal Systems	Environmental adaptation	Thermogenesis	1	339	0.7603114	9.13E-01	ko04714	XP_034336409.1	K12323
Human Diseases	Cancer: overview	MicroRNAs in cancer	1	357	0.7780466	9.13E-01	ko05206	XP_034320756.1	K05665
Organismal Systems	Endocrine system	Renin secretion	1	358	0.7789932	9.13E-01	ko04924	XP_034336409.1	K12323
Organismal Systems	Sensory system	Inflammatory mediator regulation of TRP channels	1	370	0.7900473	9.13E-01	ko04750	XP_034318676.1	K04983
Human Diseases	Neurodegenerative disease	Alzheimer disease	2	711	0.8022548	9.13E-01	ko05010	NP_001292237.1; XP_011444077.2	K21398+K21398
Organismal Systems	Circulatory system	Vascular smooth muscle contraction	1	406	0.8200525	9.17E-01	ko04270	XP_034336409.1	K12323
Human Diseases	Infectious disease: bacterial	Tuberculosis	1	433	0.839752	9.24E-01	ko05152	XP_019928009.2	K06560
Environmental Information Processing	Signal transduction	cGMP-PKG signaling pathway	1	459	0.8567133	9.27E-01	ko04022	XP_034336409.1	K12323
Human Diseases	Neurodegenerative disease	Huntington disease	1	517	0.8884503	9.33E-01	ko05016	XP_011445052.2	K05613
Human Diseases	Infectious disease: viral	Human immunodeficiency virus 1 infection	1	522	0.8908379	9.33E-01	ko05170	XP_034317200.1	K22544
Human Diseases	Neurodegenerative disease	Amyotrophic lateral sclerosis	1	616	0.9274337	9.42E-01	ko05014	XP_011445052.2	K05613
Environmental Information Processing	Signal transduction	cAMP signaling pathway	1	618	0.9280638	9.42E-01	ko04024	XP_034336409.1	K12323
Environmental Information Processing	Signaling molecules and interaction	Neuroactive ligand-receptor interaction	1	794	0.9667844	9.67E-01	ko04080	XP_011448026.2	K05194

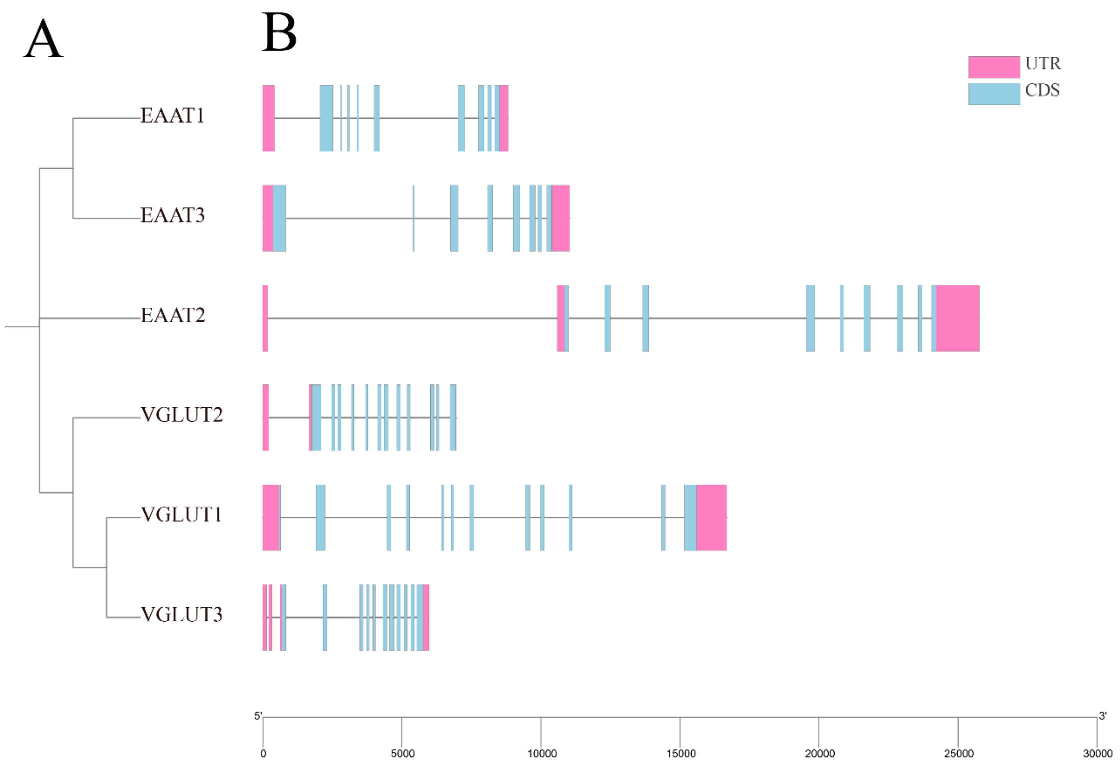

Supplementary Figure S1. Phylogenetic Tree and Gene Structure of the glutamate transporter family genes in *C. gigas*. (A) Phylogenetic tree of the glutamate transporter family in *C. gigas* (B) The black lines, pink boxes, and blue boxes represent introns, untranslated regions (UTRs), and exons, respectively.
